# Potential Cost-Effectiveness of Universal Access to Modern Contraceptives in Uganda

**DOI:** 10.1371/journal.pone.0030735

**Published:** 2012-02-17

**Authors:** Joseph B. Babigumira, Andy Stergachis, David L. Veenstra, Jacqueline S. Gardner, Joseph Ngonzi, Peter Mukasa-Kivunike, Louis P. Garrison

**Affiliations:** 1 Global Medicines Program, Department of Global Health, School of Public Health, University of Washington, Seattle, Washington, United States of America; 2 Pharmaceutical Outcomes Research and Policy Program, School of Pharmacy, University of Washington, Seattle, Washington, United States of America; 3 Departments of Epidemiology and Global Health, School of Public Health and Community Pharmacy, University of Washington, Seattle, Washington, United States of America; 4 Department of Obstetrics and Gynecology, Mbarara University of Science and Technology, Mbarara, Uganda; 5 Engender Health Fistula Care Project, Kampala, Uganda; University of Cape Town, South Africa

## Abstract

**Background:**

Over two thirds of women who need contraception in Uganda lack access to modern effective methods. This study was conducted to estimate the potential cost-effectiveness of achieving universal access to modern contraceptives in Uganda by implementing a hypothetical new contraceptive program (NCP) from both societal and governmental (Ministry of Health (MoH)) perspectives.

**Methodology/Principal Findings:**

A Markov model was developed to compare the NCP to the status quo or current contraceptive program (CCP). The model followed a hypothetical cohort of 15-year old girls over a lifetime horizon. Data were obtained from the Uganda National Demographic and Health Survey and from published and unpublished sources. Costs, life expectancy, disability-adjusted life expectancy, pregnancies, fertility and incremental cost-effectiveness measured as cost per life-year (LY) gained, cost per disability-adjusted life-year (DALY) averted, cost per pregnancy averted and cost per unit of fertility reduction were calculated. Univariate and probabilistic sensitivity analyses were performed to examine the robustness of results. Mean discounted life expectancy and disability-adjusted life expectancy (DALE) were higher under the NCP vs. CCP (28.74 vs. 28.65 years and 27.38 vs. 27.01 respectively). Mean pregnancies and live births per woman were lower under the NCP (9.51 vs. 7.90 and 6.92 vs. 5.79 respectively). Mean lifetime societal costs per woman were lower for the NCP from the societal perspective ($1,949 vs. $1,987) and the MoH perspective ($636 vs. $685). In the incremental analysis, the NCP dominated the CCP, i.e. it was both less costly and more effective. The results were robust to univariate and probabilistic sensitivity analysis.

**Conclusion/Significance:**

Universal access to modern contraceptives in Uganda appears to be highly cost-effective. Increasing contraceptive coverage should be considered among Uganda's public health priorities.

## Introduction

With a fertility rate of 6.7 and an annual population growth rate of 3.2%, Uganda has one of the fastest growing populations in the world [1]. This is due in part to low contraceptive use. Among fecund married or unmarried, sexually active women who desire contraception, only 31% use modern contraceptive methods; 61% lack access, and 8% use traditional methods [2]. Other African countries have similar contraceptive access problems. In Ethiopia for instance, only 29% of fecund married or unmarried, sexually active women who desire contraception use modern methods [3]. This results in many unintended pregnancies and unplanned births. In Uganda, 45% of births in 2006 were unplanned and women have more children per woman (6.7) than they want (5.1) [1]. More unintended pregnancies occur among non-contraceptive users (88%) than due to contraceptive failure (12%) [2].

Contraception is beneficial to individuals, families and society, and contributes to improved health and socioeconomic development [4]–[6]. But despite these potential benefits, access to contraceptives in Uganda is declining, and the government has not responded appropriately [7]. With a per capita health expenditure of US$44 (at the average exchange rate) or International$112 (purchasing power parity) [8], Uganda's government-run healthcare system must prioritize among the many competing health needs of the population because of the extreme budget constraint. Consequently, many beneficial healthcare interventions may not be implemented.

Cost-effectiveness analysis considers both costs and health outcomes in evaluating the efficiency of interventions and allows policy makers to prioritize among competing uses of healthcare resources. The objective of this study was to compare the incremental cost-effectiveness of a hypothetical new contraceptive program (NCP) that would achieve universal access to modern contraceptives in Uganda, to the current contraceptive program (CCP), i.e., the status quo in which access to modern contraception is limited. In this study, we assumed that the NCP would have an identical proportional distribution of modern contraceptive methods as is currently used in Uganda but with the unmet need for contraception removed i.e. all fecund married or unmarried, sexually active women who desire contraception, an estimated total of 3,200,000 women, use modern methods and none use traditional methods [1]. Table 1 shows the number and percent distribution of these women's use of different kinds of contraception under the CCP and the hypothetical NCP.

**Table 1:** Number and percentage of fecund married or unmarried, sexually active women in Uganda who desire contraception and the different kinds of contraceptive methods under the CCP and NCP*.

	CCP		NCP	
	n	%	n	%
All women	3,200,000	100.0	3,200,000	100.0
No contraception	1,952,000	61.0	0	0
Any method	1,248,000	39.0	3,200,000	100.0
Any modern	992,000	31.0	3,200,000	100.0
Female sterilization	108,800	3.4	352,000	11.0
Male sterilization	6,400	0.2	19,200	0.6
Pill	147,200	4.6	473,600	14.8
Intrauterine device (IUD)	6,400	0.2	19,200	0.6
Injectable	496,000	15.5	1,600,000	50.0
Implants	19,200	0.6	60,800	1.9
Male condom	204,800	6.4	659,200	20.6
Any traditional	256,000	8.0	0	0
Rhythm	124,800	3.9	0	0
Withdrawal	86,400	2.7	0	0
Folk method	44,800	1.4	0	0

CCP – Current Contraceptive Program; NCP – New Contraceptive Program.

*Assumes identical distribution of modern methods as is currently used.

## Methods

### Markov Model

**Figure 1:**
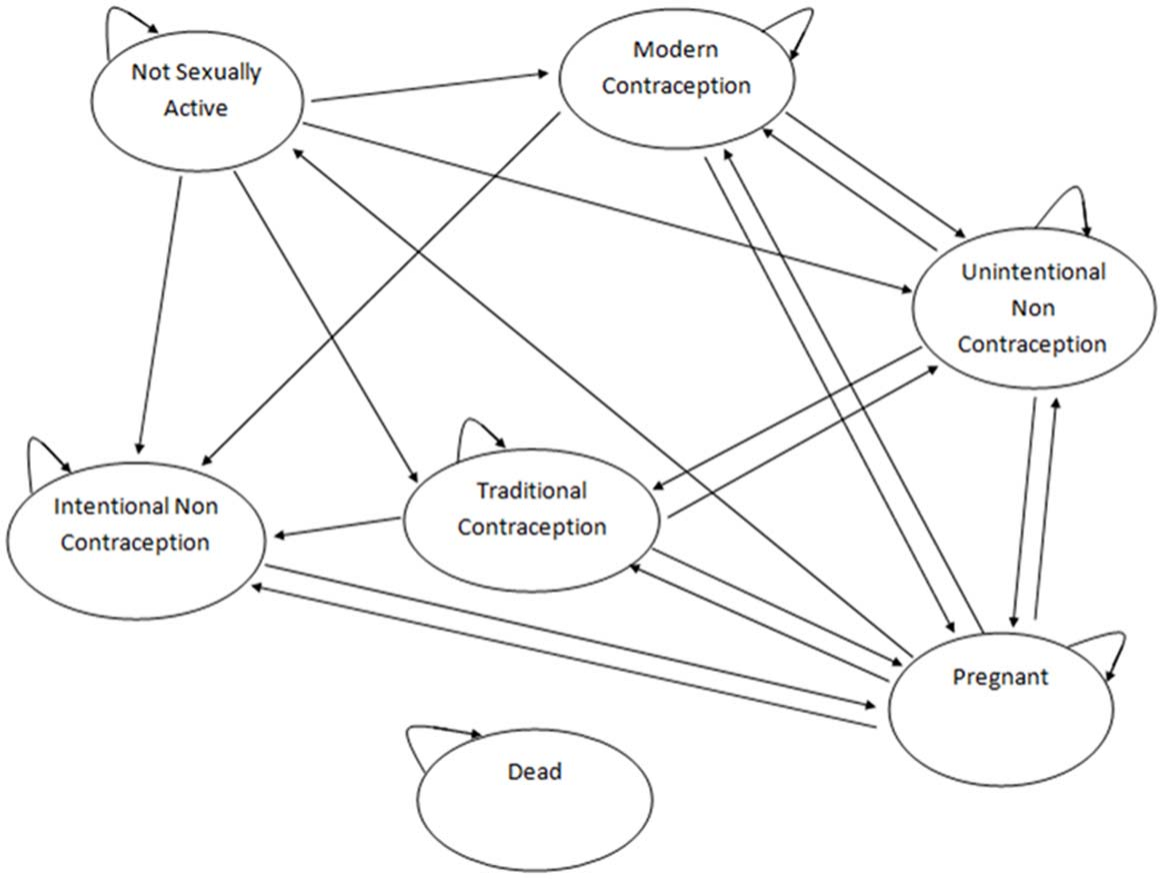
Markov model. The model illustrates the different states of contraception through which women between 15 and 49 years of age in Uganda transition. Each state is associated with a cost and a value of disability-adjusted life years lost. All states may progress to dead.

A Markov cohort model was developed to assess the potential cost-effectiveness of the NCP compared to the CCP. The model projected the reproductive health experience of a hypothetical cohort of 15-year old girls over a lifetime horizon. The starting age of the hypothetical cohort was chosen to reflect as closely as possible the median age of sexual debut in Uganda – 16.6 years [1]. Figure 1 shows a schematic of the Markov model.

The Markov model is suited to women's reproductive experience because it spans many years and many events – pregnancies, miscarriages, abortions and births – that can occur multiple times. For instance, women face multiple opportunities to get pregnant with the probability of pregnancy diminishing with each subsequent cycle as the individual ages. The model had 7 states: (i) not sexually active (NSA); (ii) intentional non-contraception (INC); (iii) unintentional non-contraception (UNC); (iv) modern contraception (MOC); (v) traditional contraception (TRC); (vi) pregnant and (vii) dead. The INC state included women who were looking to get pregnant and the UNC state included women who lacked access to modern contraception. The cycle time was 9 months. The model assumed a constant modern contraceptive use mix across all ages for women on contraception.

The model was checked (de-bugged) by varying transition probabilities between 0 and 1 to observe if responses were logical and setting costs and outcomes to 0 separately to examine if the expected values were identical. Validation was performed by comparing the predicted fertility to the published estimate for Uganda [1].

The analysis was performed from both the governmental (Ministry of Health (MoH)) and the societal perspectives and included direct and indirect costs. The MoH perspective included direct medical costs and direct non-medical costs that are incurred by the MoH which is the healthcare provider in Uganda and the societal perspective included, in addition to these, the direct non-medical costs incurred by patients (such as transportation) and indirect (productivity) costs. Costs and outcomes were discounted at 3% per year [9]. The NCP was compared to the CCP on the basis of costs, life expectancy and incremental cost-effectiveness analysis using cost per life-year (LY) saved and disability-adjusted life years (DALY) averted to capture both quality and quantity of life. The model was also used to compute other intermediate measures of cost-effectiveness: 1) cost per pregnancy averted; 2) cost per unit of fertility reduction; 3) cost per ectopic pregnancy averted; 4) cost per miscarriage averted; 5) cost per induced abortion averted; 6) cost per still birth averted 7) cost per neonatal death averted; 8) cost per infant death averted; and 9) cost per child death averted.

A threshold for cost-effectiveness with ranges from 1 to 3 times per capita GDP per DALY averted has been suggested [10]–[12] and other studies have used this threshold in Uganda [13], [14]. Uganda's GDP per capita was $474 in 2010 [15]. Therefore the NCP was judged to be highly cost-effective if the incremental cost-effectiveness ratio (ICER) was less than $474 per DALY and cost-effective if the ICER was less than $1423 per DALY (3 times per capita GDP).

### Starting distribution of the hypothetical cohort among Markov states

The proportion of 15-year olds who reported no sexual activity in the 3 months prior to the 2006 UDHS (80.3%) was started in the NSA state [1]. In the CCP, the remaining 19.7% who were sexually-active women were divided among the other states: 9.1% who used modern contraception started in the MOC state; 1.8% who used traditional contraception started in the TRC state; 6.6% who lacked access started in the UNC state; and the remaining 2.1%, considered to want to conceive, started in the INC state [1]. In the NCP, 17.6% (who started in the MOC, TRC, and the UNC states) were started in the MOC state, akin to universal access to modern contraception, and 0% was started in the INC and TRC states.

### Transition probabilities

Transition probabilities between states of contraceptive use over a woman's life were computed using UDHS data and were age-specific within five-year age intervals (Table 2) [1]. The proportion of women who remained sexually inactive represented the probability (by age group) of staying in the NSA state (UDHS 2006; Table 7.7.1 (Page 93)) [1]. Sexual activity was defined as reported sexual activity within the previous 4 weeks, consistent with the UDHS definition [1]. The proportion of women using traditional and modern contraception (UDHS 2006; Table 6.2.1 (Page 67)) [1] represented the probability (by age group) of transition between both the NSA and UNC states and MOC and TRC states respectively. The proportion of sexually active women lacking access to modern contraception (UDHS 2006; Table 7.7.1 (Page 93) [1] represented the probability (by age group) of transition between the NSA and UNC states. The proportion of sexually active women who wish to conceive was calculated by subtracting the sum of the proportions (by age group) of women who use contraceptives and women who lack access to contraceptives from 1. The resulting proportion represented the probability of transition from the NSA state to the INC state.

**Table 2:** Age-specific transition probabilities from different states of contraceptive use, pregnancy and death.

Age group	15–19	20–24	25–29	30–34	35–39	40–44	45–49
NSA→INU	0.02	0.17	0.17	0.12	0.08	0.05	0.13
NSA→UNU	0.07	0.21	0.26	0.29	0.29	0.27	0.19
NSA/UNU→MOD	0.09	0.17	0.19	0.22	0.19	0.21	0.19
NSA/UNU→TRA	0.02	0.03	0.04	0.04	0.04	0.06	0.05
NSA→NSA	0.80	0.41	0.34	0.31	0.39	0.41	0.47
UNU/INU→PRE*	0.85	0.85	0.85	0.83	0.81	0.69	0.17
UNU→to UNU	0.07	0.05	0.05	0.05	0.06	0.16	0.53
All states→Deadψ	0.002	0.003	0.006	0.009	0.012	0.011	0.011

NSA – Not Sexually Active; INU – Intentional Non-Use of contraception; UNU – Unintentional Non-Use of contraception; MOD – Modern contraception; TRA – Traditional Contraception; PRE – Pregnant.

*Initial estimate of 85% probability of pregnancy is adjusted for proportion of women who are menopausal by age.

ψ Gender and age-specific mortality rate for Uganda converted to a nine-month transitional probability.

The probability of pregnancy without contraception (85%) [16] represented the probability of transition from the UNC and INC states to the pregnant state. This probability was adjusted for the age-specific prevalence of menopause (defined as last known menstrual period occurring 6 or more months prior to survey among non-pregnant and non-amenorhoeic women (UDHS 2006; Table 7.10 (Page 98)) [1], which increases from 2.4% between 30 and 34 years to 42.8% between 48 and 49 years of age. The rate of contraceptive failure on traditional contraception (20%) and modern contraception (3%) [16] represented the probability of transition between the TRC and MOC states and pregnancy. The failure rate for modern contraceptive use was weighted by the frequency of use of different modern methods in Uganda [1].

The probability of intentional and unintentional contraceptive discontinuation, estimated in an Eastern African study at 16.7% and 12.1% respectively [17], represented the probability of transition between the MOC state and INC and the MOC and TRC states respectively, assuming that women who lose access to modern contraception opt for traditional contraception.

Women who had live births transitioned to the MOC state because the probability of pregnancy during lactation amenorrhea is similar to the probability of pregnancy on modern contraceptives [18]. Women who had non-live birth pregnancy outcomes transitioned to other states at the same rate as women in the NSA state. Transitions between the MOC and TRC states in a single cycle as well as movement from contraceptive use states to the NSA state were not allowed in a single cycle; women returned to the NSA state only after pregnancy. Pregnancy was a temporary state i.e. women did not spend more than a single cycle in this state.

All estimates reported as annual probabilities were converted into 9-month transition probabilities to reflect the cycle time of the model. The changing probability of different events such as pregnancy over time as women age (time-dependency) was captured in the model by using age-group-specific tables in lieu of the relevant transition probabilities. This enabled members of the simulated cohort of different ages to be assigned their relevant age-specific transition probabilities.

The non-age-specific transition probabilities are shown table 3.

**Table 3:** Parameters of the Markov model.

Parameter	Base case	Sensitivity range*	Reference
**Transition probabilities**			
MOD→PRE	0.03	0.02–0.03	[16]
TRA→PRE	0.20	0.16–0.24	[16]
MOD→INU	0.25	0.20–0.29	[17]
MOD→UNU	0.34	0.27–0.41	[17]
TRA→INU	0.26	0.21–0.31	[17]
TRA→UNU	0.36	0.27–0.41	[17]
PRE→NSAϕ	0.73	0.58–0.88	[1], [21], [26]–[28]
PRE→INU	0.03	0.02–0.04	[1], [21], [26]–[28]
PRE→UNU	0.06	0.05–0.08	[1], [21], [26]–[28]
PRE→MOD	0.04	0.03–0.05	[1], [21], [26]–[28]
PRE→TRA	0.01	0.01–0.02	[1], [21], [26]–[28]
PRE→Deadψ	0.0034	0.0028–0.0041	[1]
**Pregnancy Complications**			
Miscarriage	0.049	0.039–0.059	[27]
Ectopic pregnancy	0.014	0.011–0.017	[26]
Abortion	0.190	0.152–0.059	[21]
Still birth	0.017	0.014–0.020	[28]
**Mortality**			
Neonatal mortality	0.021	0.017–0.025	[1]
Infant mortality	0.055	0.044–0.067	[1]
Child mortality	0.049	0.030–0.120	[1]
Life expectancy (2.5 years)	51.7	–	[19]
**DALYs lost**			
Maternal conditions	0.272	0.218–0.327	[20]
**Costs ($US)**			
Contraception (MOH)	14.67	7.34–22.01	[22], [23]
Contraception (Societal)	64.74	32.39–97.16	[22], [23] Primary study
Pregnancy (MOH)	96.65	48.32–144.97	[22], [23]
Pregnancy (Societal)	254.33	127.13–381.49	[15], [22], [23] Primary study

*Sensitivity ranges are based on 95% confidence intervals where available or represent +/−50% for costs and +/−20% for other parameters.

ϕ Also probability of live birth. Calculated by subtracting ectopic pregancies, induced abortions, miscarriages and still births.

ψ Maternal mortality.

### Mortality

Age-specific mortality rates from all causes for women in Uganda were obtained from country-specific life tables published by the World Health Organization [19] and are shown in table 2. These were adjusted for the percentage of deaths due to maternal causes which is 13% [1]. Maternal mortality is 435 (345–524) deaths per 100,000 live births [1]. This estimate was adjusted for the proportion of pregnancies that result in live births. Neonatal, infant and child mortality estimates (table 3) were obtained from the UDHS [1] and are represented cumulatively i.e. infant mortality includes neonatal mortality and child mortality includes both neonatal and infant mortality.

### Disability-Adjusted Life Years (DALYS)

Annually, there are an estimated 498,000 DALYs lost due to maternal causes (pregnancy complications) in Uganda [20] and an estimated 1,830,000 pregnancies [21]. Therefore the average DALY loss due to pregnancy complications associated with a single pregnancy is 0.27.

### Costs

Costs were estimated for the pregnant (PRE) and modern contraception (MOC) Markov states only; we assumed that the other Markov states – NSA, INC, UNC, TRC and DEAD – bore no costs. For these two states, we estimated direct medical costs, direct non-medical costs, and indirect costs.

In the MOC state, the direct medical costs included the cost of contraceptive technology, weighted by the prevalence of the use of the different methods [22], the cost of healthcare personnel [23], transportation costs [24], costs of upkeep [24], and out-of-pocket costs when patients seek contraceptive services [23]. The direct non-medical costs included overhead costs and capital costs for out-patient care in Uganda obtained from the WHO WHOCHOICE database [25]. The indirect costs included the costs of lost time when women seek health services [24]. The costs of different contraceptive different contraceptive technologies and their prevalence of use are shown in Table S1 and the costs of other inputs are shown in Table S2.

In the PRE state, the different cost categories were estimated for antenatal care and the different potential outcomes of pregnancy – miscarriage, induced abortion, ectopic pregnancy, birth (still and live; vaginal and cesarean), obstetric hemorrhage, and eclampsia, weighted by their incidence [1], [21], [23], [26]–[28]. The direct medical costs included the costs of healthcare personnel [23] and other healthcare materials [23], transportation costs [24], costs of upkeep [24], and out-of-pocket costs when patients seek different services [23], [24]. The direct non-medical costs included the overhead and capital costs associated with different services [25] and the indirect costs included the productivity costs associated with lost time while seeking care for different services [24]. The costs of different inputs by pregnancy outcome are shown in Table S3 and the incidence of pregnancy outcomes, unit costs and total costs of pregnancy from the MoH and societal perspective are shown in Table S4. A detailed description of the estimation of the costs of modern contraception and pregnancy is given in Appendix S1.

All costs were in 2010 US dollars.

### Sensitivity Analysis

Sensitivity analyses were performed to determine which variables had substantial impact on costs and outcomes. All parameters were assigned a range of plausible values using 95% confidence intervals when available or +/−50% for costs and +/−20% for other parameters (table 2). To further test the robustness of our results, we conducted a probabilistic sensitivity analysis. We created probability distributions for all of the parameters in the model except those relating to the underlying methods of the analysis such as the discount rate [29]. For all other parameters, the base-case value was used for the mean, and the standard error was estimated based on the approximation that the range used for one-way sensitivity analyses represented a 95% confidence interval, with the range approximately equal to four times the standard error [29]. A Beta distribution was used for probabilities and DALYs because it is bounded on the interval 0–1 and resembles a normal distribution for some parameterisations and a normal distribution was used for costs because we were not overly concerned with skewness as to use a log-normal or gamma distribution [29]. Monte Carlo simulation was used to create 10,000 iterations for which the expected outcome values were calculated. The probability that either intervention was cost-effective was then calculated for a range of cost-effectiveness thresholds.

Data analysis was performed using TreeAge Pro.

## Results

### Model testing and validation

The model was tested (de-bugged) by varying transition probabilities between 0 and 1 (which resulted in logical responses) and setting costs and outcomes to 0 separately (which resulted in identical expected values). With regard to validation, we used the predicted fertility as the main benchmark and the model predicted the total fertility rate in Uganda fairly well (6.92 vs. 6.70) [1].

### Cost-consequences analysis

For a hypothetical cohort of 100,000 15-year old women, the NCP would result in savings of $3.78 million in societal costs, or $4.88 million in MoH costs. The NCP would also result in, 160,000 fewer pregnancies and 113,000 fewer births. Table 4 shows detailed results of the cost-consequences analysis including the impact on other outcomes such as abortions and ectopic pregnancies.

**Table 4:** Results of a cost-consequences analysis for a hypothetical cohort of 100,000 Ugandan women.

	CCP	NCP	Difference
Societal costs ($US)	$198,729,000	$194,940,000	−$3,789,000
MOH costs ($US)	$68,481,000	$63,603,000	−$4,878,000
Pregnancies	950,000	790,000	−160,000
Discounted Life years	2,865,000	2,874,000	9,000
Discounted DALE (years)	2,701,000	2,738,000	37,000
Ectopic pregnancies	13,300	11,100	−2,200
Induced abortions	180,000	151,000	−29,000
Miscarriages	46,000	39,000	−7,000
Still births	16,000	14,000	−2,000
Live births	692,000	579,000	−113,000
Neonatal deaths	20,000	17,000	−3,000
Infant deaths	53,000	44,000	−9,000
Child deaths	95,000	79,000	−16,000

CCP – Current Contraceptive Program; NCP – New Contraceptive Program; MoH – Ministry of Health; DALE – Disability-Adjusted Life Expectancy.

### Base case analysis

Table 5 is a summary of the baseline results of the cost-effectiveness analysis. Mean discounted life expectancy was higher under the NCP (28.74 vs. 28.65 years). The mean discounted disability-adjusted life expectancy was also higher under the NCP (27.38 vs. 27.01). The mean number of pregnancies per woman would be reduced from 9.51 under the CCP to 7.90 under the NCP, reducing the total fertility rate from 6.92 to 5.79. Other maternal and child health outcomes were also more favorable under the NCP: 0.02 fewer ectopic pregnancies, 0.07 fewer miscarriages, 0.29 fewer abortions, 0.02 fewer still births, 0.03 fewer neonatal deaths, 0.09 fewer infant deaths, and 0.16 fewer child deaths per woman on average.

**Table 5:** Results of the baseline analysis showing the mean costs (per woman), incremental costs, DALE, incremental DALE and ICERs comparing NCP to the CCP.

	CCP		NCP	
	Societal	MoH	Societal	MoH
Total cost ($)	1,987.29	684.81	1,949.40	636.03
Total incremental cost ($)			−38.89	−48.78
DALE (Years)	27.01	27.01	27.38	27.38
Incremental DALE (Years)			0.37	0.37
ICER ($/DALY)			Dominant	Dominant
Program cost	458.49	104.85	682.47	155.58
Incremental program cost			224.98	51.73
Medical cost	1,529.80	581.97	1,267.94	481.46
Averted medical costs			262.86	100.51
Net costs*			421.61	55.07

DALE – Disability-Adjusted Life Expectancy; DALY – Disability-Adjusted Life-Year; MoH – Ministry of Health; CCP – Current Contraceptive Program; NCP – New Contraceptive Program.

*Program costs minus averted medical costs.

Mean lifetime societal costs per woman were lower for the NCP from the societal perspective ($1,949 vs. $1,987) and the MoH perspective ($636 vs. $685). The NCP increased program costs from both the societal perspective (by $225) and the MoH perspective (by $51) but reduced medical costs from both the societal perspective (by $267) and the MoH perspective (by $101). Therefore the net lifetime costs (program costs minus averted medical costs) were $422 from the societal perspective and $55 from the MoH perspective.

In the incremental analysis, the NCP dominated the CCP from both the societal perspective and the MOH perspective i.e. resulted in reduced costs and more favorable outcomes.

We calculated ICERs for different outcomes (denominators) and with the costs (numerator) remaining the same. As in the baseline analysis, and from both the societal perspective and the MoH perspective, the NCP dominated the CCP i.e. resulted in reduced costs and more favorable outcomes. Table 6 summarizes the reduction in costs and improvement in a variety of outcomes comparing the NCP to the CCP.

**Table 6:** Mean incremental costs (per woman) and health outcomes comparing the new contraceptive program to the current contraceptive program in Uganda*.

Outcome (mean)	Value
Incremental societal cost ($)	−37.9
Incremental MoH cost ($)	−48.8
Incremental life expectancy (LYs)	0.09
Reduction in pregnancies	1.60
Reduction in fertility	1.13
Reduction in ectopic pregnancies	0.02
Reduction in miscarriages	0.07
Reduction in abortions	0.29
Reduction in still births	0.02
Reduction in noenatal deaths	0.03
Reduction in infant deaths	0.09
Reduction in child deaths	0.16

LYs – Life Years; ICER – Incremental Cost-Effectiveness Ratio.

*The NCP results in lower costs and improved outcomes i.e. dominates the CCP in the incremental analysis.

### Sensitivity analysis

**Figure 2:**
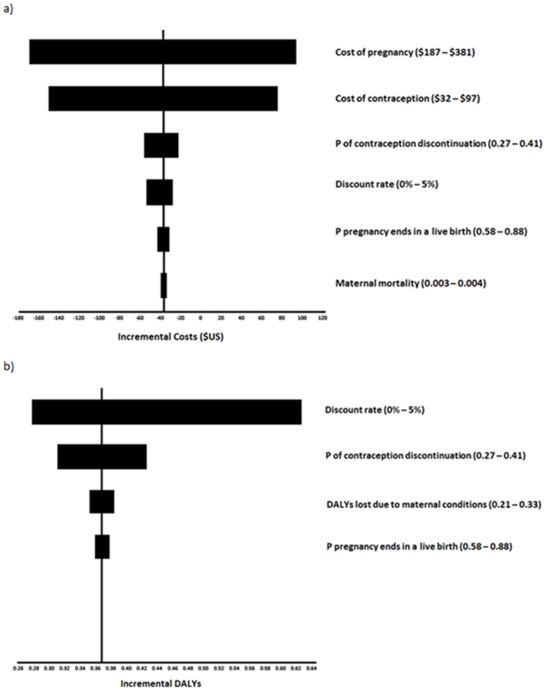
Tornado diagrams of univariate sensitivity analysis from the societal perspective. The diagram shows, for a comparison between the new contraceptive program and the current contraceptive program, the impact of uncertainty surrounding different variables on incremental cost (a) and incremental disability-adjusted life years (b). The most influential variables are shown.

**Figure 3:**
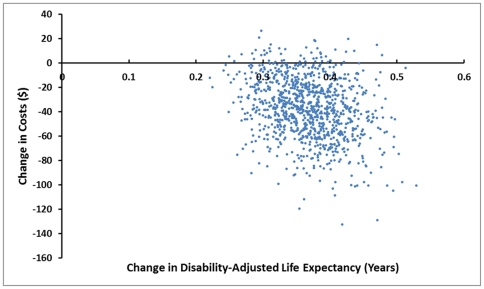
Incremental cost-effectiveness scatter plot obtained from probabilistic sensitivity analysis. The figure shows the distribution of cost-effectiveness pairs on the cost-effectiveness plane.

**Figure 4:**
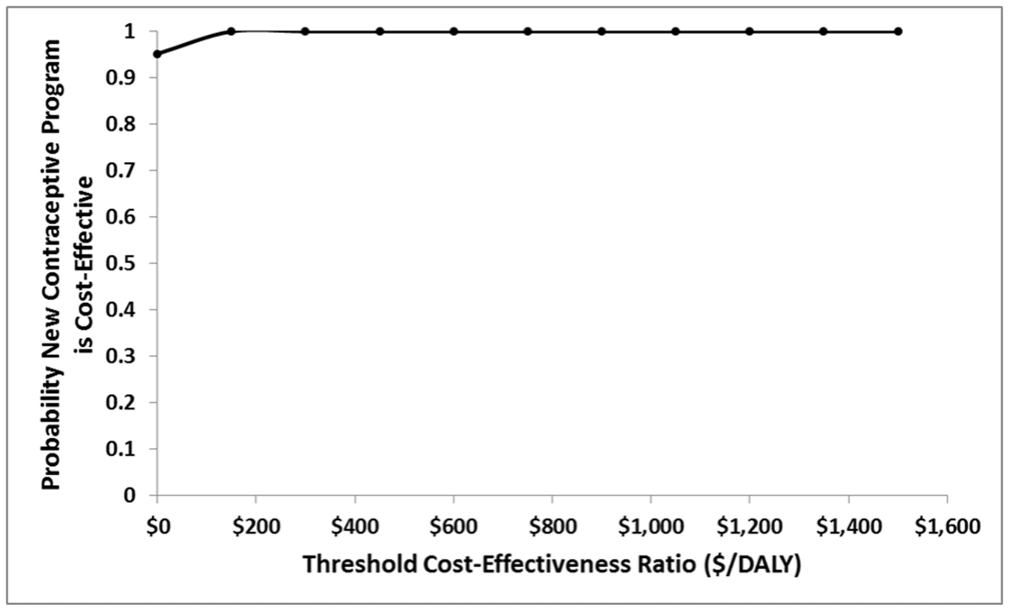
Cost-effectiveness acceptability curves obtained from probabilistic sensitivity analysis. The curves show, for 10,000 simulated samples, the probability that the new contraceptive program is cost-effective compared to the current contraceptive program at varying thresholds of cost-effectiveness (willingness to pay to avert an additional disability-adjusted life year).

Univariate sensitivity analyses (Figure 2) showed that the incremental societal cost was most sensitive to the uncertainty surrounding the costs of contraception and pregnancy. Incremental disability-adjusted life expectancy was most sensitive to the uncertainty surrounding the discount rate and the probability of modern contraception discontinuation. Probabilistic sensitivity analysis (Figure 3) showed that all cost-effectiveness pairs obtained from probabilistic sensitivity analysis lie in the “southeast” and “northeast” quadrants indicating a great deal of certainty that the NCP is more effective than the CCP and that there is some uncertainty as to whether the NCP is less costly than the CCP. Figure 4 is a cost-effectiveness acceptability curve which shows the probability that the NCP is cost-effective compared to the CCP for the range of values of willingness to pay constrained at three times Uganda's GDP per capita, a commonly used standard. It shows that the proportion of iterations in which the NCP is more cost-effective compared to the CCP approaches 100% at a cost-effectiveness threshold of much less than $474, Uganda's GDP per capita.

## Discussion

Using a Markov model, this study found that a hypothetical new contraceptive program (NCP) to achieve universal access to modern contraceptives in Uganda would be highly cost-effective, dominating the current contraceptive program (CCP) in the incremental analysis by achieving improved outcomes at a reduced cost. And findings were robust to univariate and probabilistic sensitivity analyses.

In addition to limiting the adverse health consequences of unintended pregnancies, contraception contributes towards achieving two of the eight Millennium Development Goals (MDGs) by reducing the number of unplanned births, reducing child morbidity and mortality, and increasing the resources that families and societies spend on the other, well planned children. In a country with one of the highest levels of maternal mortality in the world [1], [30], contraception could have a substantial impact on induced abortion-related morbidity and mortality in this setting of legal and religious proscriptions. The NCP would reduce the estimated 297,000 induced abortions performed annually in Uganda by almost 50,000 [21], a significant contribution given that post-abortion care, which is not illegal, is severely inadequate.

The finding that the NCP is highly cost-effective is consistent with a previous study conducted in Uganda, which found that satisfying all the unmet need for modern methods of contraception would reduce maternal mortality by 40% and unplanned births and induced abortions by 84–85% while saving $3 for every dollar invested in reducing this unmet need [2]. The present study had the added advantage of making a formal value assessment including the performance of an incremental analysis that allows for comparison with other healthcare interventions, incorporating parameter uncertainty in the modeling framework, and modeling the entire reproductive and life experience of women. And because the model was primarily based on demographic and health survey data, it can be replicated in other low-income countries with similar surveys.

In dominating the CCP, the NCP out-competes a number of health interventions that have been evaluated for cost-effectiveness in Uganda such as facility-based care for HIV ($1,396 per quality-adjusted life-year(QALY)) [13], group psychotherapy with reinforcement for depression ($1,150 per QALY) [31], home-based antiretroviral therapy compared to using septrin alone ($597 per DALY) [32], measles eradication ($284/DALY) [14], vitamin A fortification of oil ($18 per DALY) or sugar ($82 per DALY) [33], and traffic enforcement ($27 per life-year saved) [34]. Additionally, universal access to contraception would be comparatively affordable; it is projected to cost $72 million [2] compared to say, provision of facility-based care for HIV which would cost $461 million [13]. While cost-effectiveness and affordability are not the only considerations for the allocation of the scarce healthcare resources in a low-income country like Uganda, their combination is quite compelling and might lead to the most efficient use of a severely limited budget.

The trajectory of socioeconomic development is uncertain and it is unclear what the impact of universal contraceptive access would be on fertility intentions, ideal family size and preference for different methods of family planning. Empirical inquiry into these issues may be an area of potential future research to enable the setting of ongoing policy in response to the dynamic nature of the population and its maternal and child health needs. Another area of potential future research might be the improvement and adaptation of our modeling framework for use in other countries or settings as a tool to estimate the potential impact of contraceptive programs and to help in the allocation of scarce healthcare resources.

One limitation of the study was that it did not estimate the potential change in fertility preference over time and assumed constant fertility intentions. Future analyses might incorporate changing fertility intentions which are likely to follow the trajectory of socioeconomic development. Another limitation is that we did not account for a change in the use distribution of modern contraception for a new contraceptive program or over the time horizon of the analysis. Future analyses might also incorporate the likely changes in contraceptive use mix in light of the on-going development of newer, more efficacious contraceptives with fewer adverse events.

Our study was also limited because we did not measure fully the benefits of contraception. The main reason for contraception is not to improve health but to limit or regulate fertility. This has both direct health benefits and wider (indirect) health and economic benefits to society and individuals including better health and economic outcomes for well-planned children, prevention of maternal to child transmission of HIV, and productivity and labor force benefits accruing to individuals and society through improved maternal well-being. This study looked at the direct health benefits to mothers and direct maternal health costs only and likely underestimates the potential cost-effectiveness of increasing contraceptive coverage. Future studies might explore the potential economic impact of a wider array of benefits, particularly impacts on child health.

In summary, universal access to modern contraception appears to be highly cost-effective and would contribute directly to achieving MDGs 4 (reduce child mortality) and 5 (improve maternal health). It would also contribute indirectly to MDG 1 (eradicate extreme hunger and poverty), MDG 2 (achieve universal primary education), and MDG 3 (promote gender equality and empower women). Therefore, policy makers in the national ministry of health and other stakeholders and development partners should consider urgent, concrete steps to increase access to modern contraceptives to women in Uganda who need them.

## Supporting Information

Table S1 **Costs of different contraceptives technologies and their prevalence of use.**
(DOCX)

Table S2 **Costs of other contraceptive inputs.**
(DOCX)

Table S3 **Costs of different inputs by pregnancy outcome.**
(DOCX)

Table S4 **Incidence of pregnancy outcomes, unit costs and total costs of pregnancy from the Ministry of Health (MoH) and societal perspectives.**
(DOCX)

Appendix S1 **Calculation of the Costs of the Contraception and Pregnancy Markov States.**
(DOCX)
