# Supplementary material for: Potential Cost-Effectiveness of Universal Access to Modern Contraceptives in Uganda
Source: PLoS One. 2012 Feb 17;7(2):e30735. doi: 10.1371/journal.pone.0030735 (PMC3281877; doi:10.1371/journal.pone.0030735)
Supplement: Table S1 — Costs of different contraceptives technologies and their prevalence of use. (DOCX) [file pone.0030735.s001.docx]

Table S1 – Costs of different contraceptives technologies and their prevalence of use

| Item | Unit | Unit cost | Cost |
| --- | --- | --- | --- |
| **Contraceptive technology** |  |  |  |
| Oral contraceptive pill | 35% | $6.05 | $2.12 |
| Depo Provera | 45% | $5.42 | $2.44 |
| Condom | 10% | $5.74 | $0.57 |
| Intra Uterine Device | 4% | $1.66 | $0.07 |
| Norplant | 1% | $34.60 | $0.35 |
| Sterilization | 5% | $4.95 | $0.25 |
| Total | 100% |  | $5.79 |
