# Supplementary material for: Potential Cost-Effectiveness of Universal Access to Modern Contraceptives in Uganda
Source: PLoS One. 2012 Feb 17;7(2):e30735. doi: 10.1371/journal.pone.0030735 (PMC3281877; doi:10.1371/journal.pone.0030735)
Supplement: Table S2 — Costs of other contraceptive inputs. (DOCX) [file pone.0030735.s002.docx]

Table S2 – Costs of other contraceptive inputs

| **Cost category** | Visits/Cycle | Unit cost | Cost | Source |
| --- | --- | --- | --- | --- |
| Personnel | 3 | $1.01 | $3.03 | [2] |
| Overhead and capital | 3 | $1.95 | $5.85 | [3] |
| Out-of-pocket | 3 | $1.48 | $4.44 | [2] |
| Travel | 3 | $2.49 | $7.47 | [2] |
| Upkeep | 3 | $11.59 | $34.77 | [2] |
| Productivity loss | 3 | $1.14 | $3.42 | [2] |
