# Supplementary material for: Potential Cost-Effectiveness of Universal Access to Modern Contraceptives in Uganda
Source: PLoS One. 2012 Feb 17;7(2):e30735. doi: 10.1371/journal.pone.0030735 (PMC3281877; doi:10.1371/journal.pone.0030735)
Supplement: Table S3 — Costs of different inputs by pregnancy outcome. (DOCX) [file pone.0030735.s003.docx]

Table S3 – Costs of different inputs by pregnancy outcome

| **Category (Reference)** | **Antenatal**  **Care** | **Vaginal Delivery** | **Cesarean**  **Delivery** | **Hemorrhage** | **Eclampsia** |
| --- | --- | --- | --- | --- | --- |
| Personnel[2, 4] | $5.39 | $14.13 | $22.87 | $56.61 | $150.21 |
| Other medical materials[3, 4] | $12.35 | $7.65 | $91.83 | $78.27 | $32.93 |
| Indirect (Overhead and capital)[2, 4] | $9.36 | $20.76 | $45.38 | $31.11 | $59.66 |
| Out-of-pocket[2, 4] | $4.45 | $1.48 | $1.48 | $1.48 | $1.48 |
| Travel[2] | $7.47 | $2.49 | $2.49 | $2.49 | $2.49 |
| Upkeep[2] | $34.77 | $11.59 | $11.59 | $11.59 | $11.59 |
| Prod loss (seeking treatment)[2] | $3.42 | $23.76 | $23.76 | $23.76 | $23.76 |
| **Total (Societal)** | **$77.22** | **$121.38** | **$238.93** | **$244.83** | **$321.64** |
| **Total (MoH)** | **$27.10** | **$42.54** | **$160.09** | **$165.99** | **$242.80** |
