# Supplementary material for: Potential Cost-Effectiveness of Universal Access to Modern Contraceptives in Uganda
Source: PLoS One. 2012 Feb 17;7(2):e30735. doi: 10.1371/journal.pone.0030735 (PMC3281877; doi:10.1371/journal.pone.0030735)
Supplement: Table S4 — Incidence of pregnancy outcomes, unit costs and total costs of pregnancy from the Ministry of Health (MoH) and societal perspectives. (DOCX) [file pone.0030735.s004.docx]

Table S4 – Incidence of pregnancy outcomes, unit costs and total costs of pregnancy from the MoH and societal perspectives

| Pregnancy outcome | Incidence  (Source) | Unit cost  (MoH) | Unit cost  (Societal) | Mean cost  (MoH) | Mean cost  (Societal) |
| --- | --- | --- | --- | --- | --- |
| Antenatal Care | 1.000 (Assumption) | $27.10 | $77.22 | $27.10 | $77.22 |
| Miscarriage | 0.049 [5] | $14.00 | $177.40 | $0.69 | $8.69 |
| Induced abortion | 0.190 [6] | $14.00 | $177.40 | $2.66 | $33.71 |
| Ectopic pregnancy | 0.014 [7] | $165.99 | $244.83 | $2.32 | $3.43 |
| Vaginal still birth | 0.014 [5, 8] | $42.54 | $121.38 | $0.61 | $1.74 |
| Cesarean still birth | 0.003 [5, 8] | $160.09 | $238.93 | $0.43 | $0.64 |
| Vaginal live birth | 0.615 [5] | $42.54 | $121.38 | $26.18 | $74.70 |
| Cesarean live birth | 0.115 [5] | $160.09 | $238.93 | $18.35 | $27.38 |
| Obstetric Hemorrhage | 0.103 [7] | $165.99 | $244.83 | $17.10 | $25.22 |
| Eclampsia | 0.005 [7] | $242.80 | $321.64 | $1.21 | $1.61 |
| **Total** |  |  |  | **$96.65** | **$254.33** |
