# Supplementary material for: Potential Cost-Effectiveness of Universal Access to Modern Contraceptives in Uganda
Source: PLoS One. 2012 Feb 17;7(2):e30735. doi: 10.1371/journal.pone.0030735 (PMC3281877; doi:10.1371/journal.pone.0030735)
Supplement: Appendix S1 — Calculation of the Costs of the Contraception and Pregnancy Markov States. (DOCX) [file pone.0030735.s005.docx]

PONE-D-11-09423

**Potential Cost-Effectiveness of Universal Access to Modern Contraceptives in Uganda**

Joseph B. Babigumira, Andy Stergachis, David L. Veenstra, Jacqueline S. Gardner, Joseph Ngonzi, Peter Mukasa-Kivunike, and Louis P. Garrison

Supplementary Appendix: Calculation of the Costs of the Contraception and Pregnancy Markov States

**Estimation of the costs of contraception**

To estimate the costs of contraception, we obtained data on the cost of 9 months of contraception (to equal the cycle time used in the model) for the different contraceptive methods (in 2010 $US) and weighted these estimates by the prevalence of use of the different methods.[1] These estimates are shown in table S1.

Table S2 shows the cost of other contraceptive inputs as well as data sources. The estimates assume quarterly visits for contraceptive services i.e. three quarters per analytic cycle.

The sum of contraceptive technology costs, personnel costs and overhead and capital costs constitute the cost of contraception from a Ministry of Health (MoH) perspective ($14.67). When these are added to out-of-pocket costs, upkeep costs and costs of lost productivity while seeking care, the result is the societal cost of contraception ($64.77). These estimates appear in table 3 in the main manuscript.

**Estimation of the costs of pregnancy**

To estimate costs, we divided pregnancy into its different potential outcomes and weighted their costs by their incidence of occurrence on average. The following outcomes were considered: antenatal care, miscarriage, abortion, ectopic pregnancy, vaginal delivery, cesarean delivery and complicated delivery (eclampsia and obstetric hemorrhage).

We performed an estimation of the costs of antenatal care (assuming 3 visits per pregnancy), vaginal delivery, cesarean delivery, obstetric hemorrhage and eclampsia. We obtained the cost of abortion from a previous study[3] and assumed that the cost of miscarriage is identical to the cost of abortion. We assumed that the cost of ectopic pregnancy is equal to the cost of obstetric hemorrhage. Table S3 shows the different cost categories, their values and data sources for the costs of antenatal care, vaginal delivery, cesarean delivery, obstetric hemorrhage and eclampsia. The total costs from table S3 were applied to the incidence of occurrence of the different outcomes of pregnancy, shown in table S4 with data sources.

The total costs of pregnancy from a MoH perspective ($96.65) and the societal perspective (254.33) appear in table 3 of the main manuscript.

**References**

1. Weissman E, Sentumbwe-Mugisa O, Mbonye AK, Kayaga E, Kihuguru SM, Lissner C (1999) Ministry of Health Uganda; World Health Organization. Safe Motherhood Program Costing Study. Accessed at <http://www.who.int/reproductivehealth/publications/maternal_perinatal_health/RHR_99_9/en/index.html> on August 13th 2010.

2. Babigumira JB, Stergachis A, Veenstra DL, Gardner JS, Ngonzi J, et al. (2011) Estimating the Costs of Induced Abortion in Uganda: A Model-Based Analysis. BMC Public Health 11: 904.

3. Choosing Interventions that are Cost-Effective. Estimates of Unit Costs of Patient Services in Uganda Accessed at [http://www.who.int/choice/country/uga/cost/en/index.html on July 20th 2010].

4. Levin A, Dmytraczenko T, McEuen M, Ssengooba F, Mangani R, et al. (2003) Costs of maternal health care services in three anglophone African countries. Int J Health Plann Manage 18: 3-22.

5. Casterline JB (1989) Collecting data on pregnancy loss: a review of evidence from the World Fertility Survey. Stud Fam Plann 20: 81-95.

6. Singh S, Prada E, Mirembe F, Kiggundu C (2005) The incidence of induced abortion in Uganda. Int Fam Plan Perspect 31: 183-191.

7. Liskin LS (1992) Maternal morbidity in developing countries: a review and comments. Int J Gynaecol Obstet 37: 77-87.

8. Stanton C, Lawn JE, Rahman H, Wilczynska-Ketende K, Hill K (2006) Stillbirth rates: delivering estimates in 190 countries. Lancet 367: 1487-1494.
